# Comparative Genomic Analysis of *Bifidobacterium bifidum* Strains Isolated from Different Niches

**DOI:** 10.3390/genes12101504

**Published:** 2021-09-25

**Authors:** Wenwei Lu, Zhangming Pei, Mengning Zang, Yuan-kun Lee, Jianxin Zhao, Wei Chen, Hongchao Wang, Hao Zhang

**Affiliations:** 1 State Key Laboratory of Food Science and Technology, Jiangnan University, Wuxi 214122, China; luwenwei@jiangnan.edu.cn (W.L.); 7190112086@stu.jiangnan.edu.cn (Z.P.); 6190111125@stu.jiangnan.edu.cn (M.Z.); jianxinzhao@jiangnan.edu.cn (J.Z.); chenwei66@jiangnan.edu.cn (W.C.); 2 School of Food Science and Technology, Jiangnan University, Wuxi 214122, China; 3 National Engineering Research Center for Functional Food, Jiangnan University, Wuxi 214122, China; 4 Department of Microbiology & Immunology, Yong Loo Lin School of Medicine, National University of Singapore, Singapore 117597, Singapore; micleeyk@nus.edu.sg; 5 Wuxi Translational Medicine Research Center and Jiangsu Translational Medicine Research Institute Wuxi Branch, Wuxi 214122, China

**Keywords:** *Bifidobacterium bifidum*, comparative genomics, diversity, genotype

## Abstract

The potential probiotic benefits of *Bifidobacterium bifidum* have received increasing attention recently. We used comparative genomic analysis to explore the differences in the genome and the physiological characteristics of *B. bifidum* isolated from the fecal samples of Chinese adults and infants. The relationships between genotypes and phenotypes were analyzed to assess the effects of isolation sources on the genetic variation of *B. bifidum*. The phylogenetic tree results indicated that the phylogeny of *B. bifidum* may be related to the geographical features of its isolation source. *B. bifidum* was found to have an open pan-genome and a conserved core genome. The genetic diversity of *B. bifidum* is mainly reflected in carbohydrate metabolism- and immune/competition-related factors, such as the glycoside hydrolase gene family, bacteriocin operons, antibiotic resistance genes, and clustered regularly interspaced short palindromic repeats (CRISPR)-Cas. Additionally, the type III A CRISPR-Cas system was discovered in *B. bifidum* for the first time. *B. bifidum* strains exhibited niche-specific characteristics, and the results of this study provide an improved understanding of the genetics of this species.

## 1. Introduction

*Bifidobacterium bifidum* is a bifidobacterial species with a high prevalence in the human gut microbiome [1]. It is among the earliest bacterial species to colonize the intestinal tract of newborns [2]. It is generally believed that *B. bifidum* present in the intestines of infants may originate from vertical maternal transmission [3], and its predominance in the intestinal tract of breastfed infants is due to its capacity to utilize specific host-derived glycans, such as human milk oligosaccharides [4,5] and mucin [6]. The beneficial effects of *B. bifidum* include restoring injured intestinal mucosa [7], modulating perturbation and the gut metabolic profile [8], suppressing colitis [9], reducing cholesterol levels [10], and ameliorating spatial memory impairment [11]. Therefore, *B. bifidum* has powerful probiotic functions and the potential to prevent or treat a range of human diseases. It is currently available as a functional food ingredient, and it can also be exploited as a therapeutic [12].

In recent years, the genomes of many bifidobacterial strains, especially those used in the probiotic industry, have been sequenced [13]. As of December 2020, more than 100 *B. bifidum* genome assemblies have been deposited in the National Center for Biotechnology Information (NCBI) GenBank database, and approximately two-thirds of these were newly sequenced in the past three years, indicating that *B. bifidum* genomics is a hot research topic. Turroni et al. [6] studied the mechanism of mucin metabolism in *B. bifidum* PRL2010 and found that a set of chromosomal loci that encode specific mucin-degrading enzymes is prevalent in *B. bifidum*. Kim et al. [14] evaluated the safety of *B. bifidum* BGN4, in terms of genome stability, antibiotic resistance gene (ARG) transferability, and the presence of virulence factors. Zhurina et al. completely annotated the genome sequence of *B. bifidum* S17, which was shown to strongly adhere to intestinal epithelial cells and has potent anti-inflammatory activity in vitro and in vivo [15]. Gueimonde et al. reported the genome sequences of *B. bifidum* LMG13195, which can interact with human immune cells, generating functional regulatory T cells [16]. Andryuschenko et al. described the draft genome sequence of *B. bifidum* ICIS-310, isolated from the feces of a healthy 5-year-old child from Orenburg [17].

With an increasing number of available genomes for a single species, comparative genomics techniques are frequently used to analyze the genetic background, habitat adaptability, and evolutionary changes of bacterial species. Many comparative genomics studies have been published for common bifidobacterial species, such as *B. longum*, *B. breve*, *B. animalis*, and *B. adolescentis* [18,19,20,21]. Several studies have depicted the genetic diversity of *B. bifidum* strains using random amplified polymorphic DNA (RAPD) [22] and multilocus sequence typing (MLST) [23]. In contrast, there is only one comparative genomics study of *B. bifidum* [13]. In that study, Duranti et al. sequenced the genomes of 15 *B. bifidum* strains and proposed that *B. bifidum* harbors a closed pan-genome and a conserved core genome. The ecological adaptability of *B. bifidum* was also noted, based on its utilization of host-derived glycans [13]. However, we propose that these findings need to be verified by analyzing additional *B. bifidum* genomes. Furthermore, the *B. bifidum* genomes that have been sequenced thus far are predominantly from Europe or the USA. To systematically analyze the genomic diversity and phylogenetic evolution of *B. bifidum*, and to extend our knowledge of other aspects of this species, it is crucial to expand the dataset with strains isolated from different regions.

In this study, we isolated 85 *B. bifidum* strains from the fecal samples of Chinese adults or infants. We sequenced the genomes of these strains and assembled them into scaffolds. The average nucleotide identity (ANI) was then calculated, the pan-genome and core genome were analyzed, and a phylogenetic tree was constructed for 140 *B. bifidum* genomes (including 55 reference assembly sequences obtained from GenBank). These data provided a more detailed and reliable basis for assessing the genome openness and genetic diversity of *B. bifidum*. We then identified the CRISPR-Cas systems, prophage fragments, and bacteriocin genes in all 140 *B. bifidum* genomes, resulting in invaluable information about the genome composition, mobile elements, and defense systems of the species. Finally, a comprehensive scanning of carbohydrate-active enzymes (CAZys) and ARGs was performed. Experiments investigating the carbohydrate utilization capacity and antibiotic resistance of the 85 isolated strains were performed to identify potential genotype-phenotype associations in *B. bifidum*.

## 2. Materials and Methods

### 2.1. Bacterial Strain Screening

The fecal samples from Chinese adults and infants used in this study were previously collected by our team and were randomly selected for strain screening. After appropriate serial dilution, each sample was spread on DeMan-Rogosa-Sharpe (MRS) agar culture medium supplemented with 0.05% (*w*/*v*) L-cysteine hydrochloride and incubated at 37 °C for 24–48 h in a chamber with an anaerobic atmosphere (10% [*v*/*v*] H_2_, 10% [*v*/*v*] CO_2_ and 80% [*v*/*v*] N_2_). Several colonies were picked from each plate and streaked on MRS medium to obtain pure colonies. The final pure culture was incubated at 37 °C for 24 h under anaerobic conditions, and preserved in 30% (*w*/*v*) glycerol at −80 °C. The species of all strains were preliminarily determined using 16S rRNA-based identification (GENEWIZ Co., Ltd., Suzhou, China). All reagents used in the processes described above were purchased from Sinopharm Chemical Reagent Co., Ltd. (Shanghai, China).

### 2.2. DNA Extraction, Genome Sequencing, Assembly, and Annotation

All *B. bifidum* strains were cultured in liquid MRS medium and incubated at 37 °C for 24–48 h. The bacterial culture medium was centrifuged at 6000× *g* rpm for 3 min, and then the supernatant was discarded. Next, bacterial cells were washed in 0.9% sterile normal saline and collected by centrifugation (6000× *g* rpm, 3 min). The rapid bacterial genomic DNA isolation kit (Sangon Biotech Ltd., Shanghai, China) was used for genomic DNA extraction.

*B. bifidum* genomes were sequenced at Novogene BioTech Co. (Tianjin, China) or Majorbio BioTech Co., Ltd. (Shanghai, China) using an Illumina Hiseq X Ten platform (San Diego, CA, USA). DNA samples that passed quality control were used for library construction, which yielded fragments of approximately 400 bp for paired-end sequencing. Single-end sequencing yielded a read length of 150 bp, and raw data from each sample had no less than 100-fold genomic coverage. Sequence reads were assembled using SOAPdenovo v2.04 software [24], and the optimized sequence is spliced under multiple Kmer parameters to obtain the optimal assembly result. Local inner gaps filling and base correction were performed using the software GapCloser v1.12 [25]. CheckM (https://ecogenomics.github.io/CheckM/, accessed on 30 May 2020) [26] was used to evaluate the quality of assembly genomes. Protein-encoding open reading frames (ORFs) were predicted using Glimmer 3.02 [27]. Functional assignments were performed and manually edited based on similarity searches against a nonredundant protein database provided by the NCBI and the Cluster of Orthologous Groups protein database. Specific information on the strains and the quality data of each genome (genome size, GC content, genome level, number of scaffolds, and N_50_ value) are listed in Table S1.

### 2.3. ANI Calculation, Pan-/Core Genome Analysis, Orthologous Gene Clustering, and Phylogenetic Tree Construction

The pairwise ANI values of *B. bifidum* genomes were calculated and visualized using the PYANI v0.2.9 package (https://github.com/widdowquinn/pyani/, accessed on 15 November 2020). Pan-genome and core-genome profiles were constructed using the PGAP v1.2.1 package [28], and all genes were divided into the core genome and the dispensable genome. Orthologous gene clustering and Venn diagram construction were performed using ORTHOMCL v2.0.9 [29]. All parameters were kept as default.

The orthologous genes of all *B. bifidum* genomes were aligned using MAFFT v7.3 [30]. Phylogeny Inference Package v3.6 was used to construct the phylogenetic tree, based on the aligned orthologous genes, using the neighbor-joining method. The final phylogenetic tree was visualized using the webserver EvolView v3 [31].

### 2.4. Prediction of the CRISPR-Cas System, Prophages, and Bacteriocin Operons

CRISPR arrays and repeat sequences were identified using the CRISPRCasFinder program (https://crisprcas.i2bc.paris-saclay.fr/CrisprCasFinder/Index accessed on 15 November 2020) [32]. MacSyFinder [33] was used to scan the corresponding Cas genes and determine the type/subtype of the CRISPR-Cas system [33]. The secondary structure of CRISPR repeats was predicted using the RNAfold web server (http://rna.tbi.univie.ac.at//cgi-bin/RNAWebSuite/RNAfold.cgi/, accessed on 20 December 2020) [34]. The phylogenetic tree, based on the CRISPR repeats, was established using MEGA X [35]. PHASTER (http://phaster.ca/, accessed on 2 October 2020) [36] was used to identify prophage fragments in the *B. bifidum* genomes. Bacteriocin operons were identified and visualized using the BAGEL4 web server (http://bagel4.molgenrug.nl/index.php/, accessed on 5 November 2020) [37]. All the above predictions were performed with default parameters supplied by the websites.

### 2.5. CAZy Scanning and Determination of Carbohydrate Utilization Capacity

The genomes of all 85 isolated *B. bifidum* strains were aligned against the CAZy database (http://www.cazy.org/, accessed on 25 November 2020) [38], and a conservative threshold (amino acid identity ≥ 30%, E-value ≤ 1 × 10^−5^) was used to predict putative CAZys. CAZys that were identified in this study included glycoside hydrolases (GHs), glycosyltransferases (GTs), carbohydrate esterases (CEs), and carbohydrate-binding modules (CBMs).

All 85 *B. bifidum* strains were precultured anaerobically in MRS liquid medium at 37 °C for 24 h. Bacterial cells were then collected by centrifugation at 8000× *g* for 3 min, washed, and resuspended in sterile normal saline. All strains were inoculated with 2% (*v*/*v*) inoculum, into a series of MRS liquid media in which different carbohydrates were used as the sole carbon source. An MRS liquid medium with glucose and a carbohydrate-free liquid medium was used as the positive and negative controls, respectively. Carbohydrate utilization capacity tests were performed to evaluate seven carbohydrate sources, including lactose, 2′-fucosyllactose, mucin, sucrose, fructo-oligosaccharide, inulin, and soluble starch. A bromocresol purple solution (0.5%, *w*/*v*) was added to the medium at 1.5% (*v*/*v*) as an indicator, and growth status was determined based on the color change in the medium. A change from purple (unavailable) to yellow (available) indicated the growth and acid production levels of the strain.

### 2.6. ARG Prediction and Antibiotic Susceptibility Testing

*B. bifidum* genomes were aligned against sequences from the latest version of the Comprehensive Antibiotic Resistance Database [39], and a conservative threshold (amino acid identity ≥ 30%, comparison hit-bit score ≥ 37.0) was used to predict putative ARGs.

The antibiotic susceptibility of the *B. bifidum* strains was evaluated using the broth microdilution method, according to ISO 10932:2010 [40]. The following 10 antibiotics were tested: tetracycline, erythromycin, clindamycin, ampicillin, amoxicillin, trimethoprim, ciprofloxacin, chloramphenicol, rifampicin, and vancomycin (all purchased from Sangon Biotech Co., Ltd., Shanghai, China). The microbiological breakpoints of *Bifidobacterium* recommended by the European Food Safety Authority were used to distinguish susceptible strains from resistant strains.

### 2.7. Data Visualization and Statistical Analysis

Line charts, violin plots, and histograms were constructed using Prism v8.0 (GraphPad, San Diego, CA, USA). The heatmaps of CAZys, carbohydrate utilization capacity, and ARGs were constructed using HemI v1.0 [41]. A Mann-Whitney U test was performed using SPSS PASW Statistics v18.0 (IBM, Armonk, NY, USA).

### 2.8. Data Availability

All 85 *B. bifidum* genomes sequenced in this study have been deposited in the NCBI GenBank database under project no. PRJNA681061. The accession numbers of all 140 sequences (including 55 reference *B. bifidum* genome sequences downloaded from GenBank) are listed in Table S1.

## 3. Results

### 3.1. General Features and ANI Values of B. bifidum

**Figure 1:**
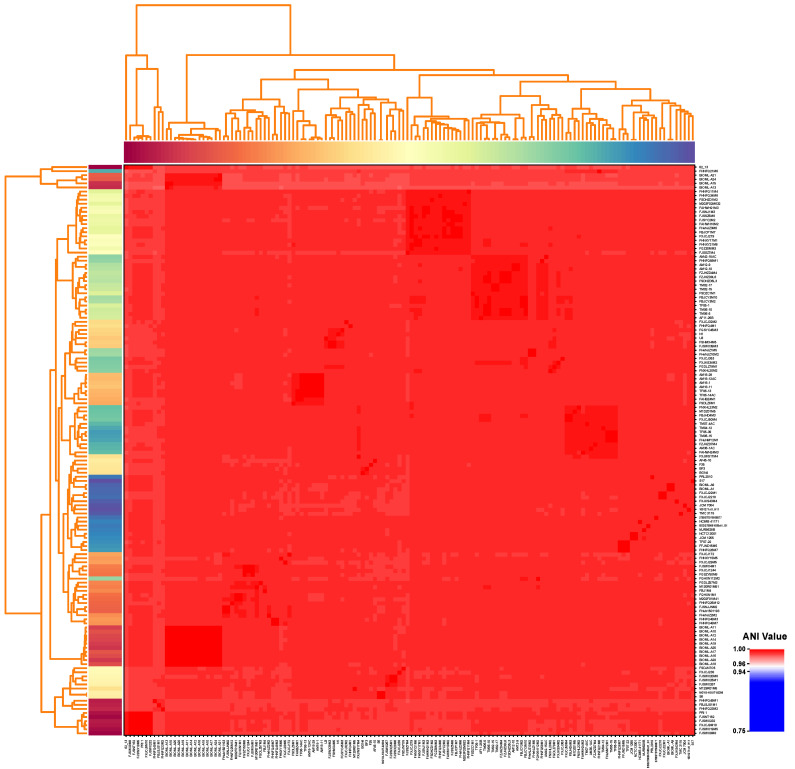
Pairwise average nucleotide identity values across 140 *B. bifidum* genomes. The color coding for the genomes on the *x*-axis and *y*-axis was used to differentiate the strains.

We obtained more than 100 *B. bifidum* isolates from 85 samples in this study. One strain from each independent sample was then selected for draft-genome sequencing. Of these 85 *B. bifidum* strains, 68% (58/85) were isolated from infant feces, and the other 27 were isolated from adult feces. The genomes of these 85 *B. bifidum* strains were subjected to further analysis, along with another 55 publicly available *B. bifidum* reference genomes. Specific information on all 140 strains/genomes is given in Table S1. The genome sizes of most *B. bifidum* strains were 2.03–2.55 Mb, with an average of 2.17 ± 0.09 Mb, and each genome contained an average of 1837 ± 143 ORFs. However, a unique sequence was identified in *B. bifidum* 62_13, which had a genome size of only 1.68 Mb, which encoded the smallest number of ORFs (1,365). The guanine and cytosine content (G+C content) of *B. bifidum* was relatively stable, ranging from 62.3% to 62.8%. The pairwise ANI values of *B. bifidum* genomes ranged from 97.73% to 99.99% (Figure 1), which was greater than the threshold of 96%. This verified that all 140 strains, including *B. bifidum* 62_13, were the same species, without any subspecies. Moreover, the relatively high ANI values indicated only minor differences between *B. bifidum* genomes. The ANI threshold for differentiating strains within the same species was updated to 96% [42]. Due to the small difference between our inter-cluster ANI and the accepted threshold, we speculated that there are minor differences between *B. bifidum* genomes.

### 3.2. Pan-Genome and Core Genome of B. bifidum

**Figure 2:**
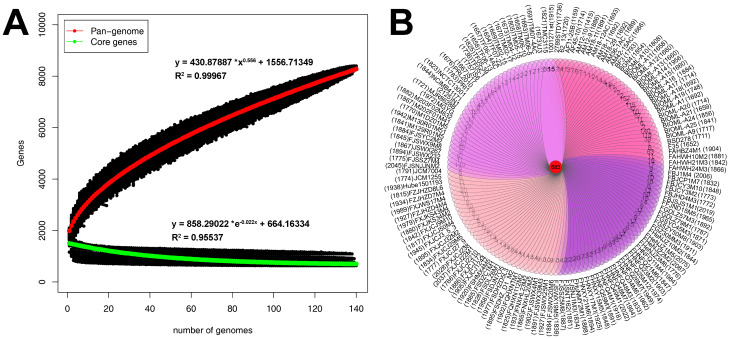
Pan-genome and core genes of 140 *B. bifidum* genomes (**A**). The 683 core genes and unique genes among *B. bifidum* genomes (**B**).

To determine the total number of different genes and the number of conserved genes present in representative *B. bifidum* strains, we performed pan-genome and core genome analyses of the 140 *B. bifidum* genomes. The number of core genes and pan-genes and the number of sequenced strains were used to construct a functional relationship diagram. The results showed that the pan-genome of all 140 strains of *B. bifidum* consisted of 8399 genes. The pan-genome asymptotic curve did not reach a plateau (Figure 2A), suggesting that when more *B. bifidum* genomes are identified with novel genes, the pan-genome would continuously increase. Meanwhile, the exponential value of the deduced mathematical function was >0.5 (Figure 2A). These findings indicate that *B. bifidum* had an open pan-genome. In contrast, the core genome demonstrated a power trend line that plateaued. This was represented by 638 genes and accounted for approximately 35% of the total number of gene families. The Venn diagram showed the presence of 683 homologous genes among all 140 *B. bifidum* genomes, while the specific number of genes for each genome ranged from 1 to 157 (Figure 2B). When the core genes were classified according to their function, the major functional category identified was associated with the utilization of host-derived glycans. These genes allow *B. bifidum* to break down host-derived glycans, such as human milk oligosaccharides and mucin, and they are closely related to the frequent localization of *B. bifidum* in the intestinal tract of infants. Another common functional category was associated with gene transcriptional regulators. *B. bifidum* was also found to harbor two core genes encoding an ABC-type dipeptide transport system, which is related to the quorum-sensing pathway.

Furthermore, to evaluate the contribution of the genomes sequenced in this study to the genetic diversity of *B. bifidum*, we performed the same analysis on 55 *B. bifidum* reference genomes. This analysis showed that there were 4602 genes in the pan-genome, 669 genes in the core genome (Figure S1A), and 707 homologous genes (Figure S1B). Based on the results of the two analyses, the sequencing data from this study greatly increased our knowledge of the genetic diversity of *B. bifidum* and confirmed that *B. bifidum* has an open pan-genome and a conserved core genome.

### 3.3. Phylogenetic Analysis of B. bifidum

**Figure 3:**
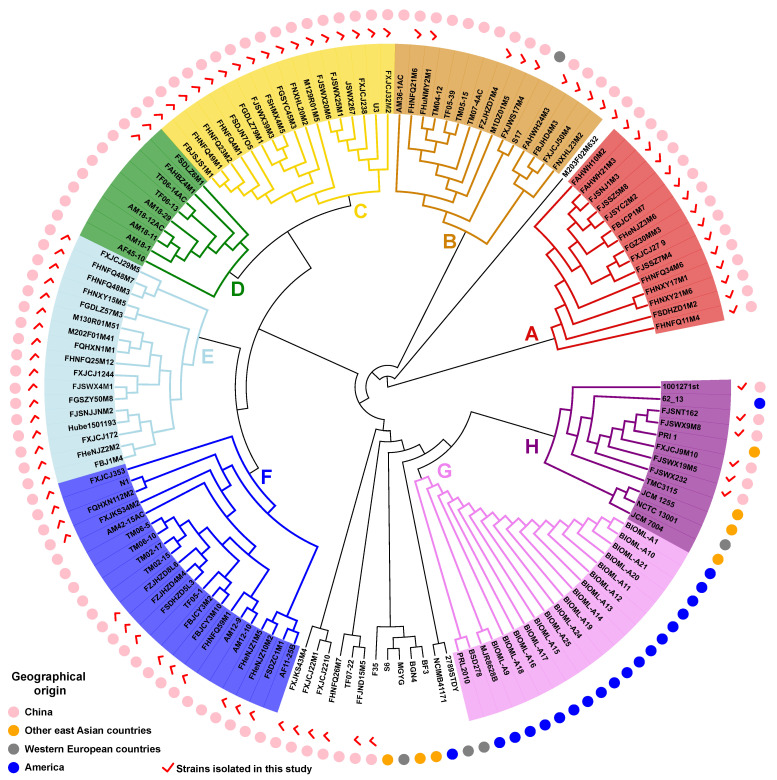
The phylogenetic tree of 140 *B. bifidum* genomes based on orthologous genes.

To further explore the evolution and genetic variation of *B. bifidum*, we constructed a phylogenetic tree based on the homologous genes of 140 *B. bifidum* genomes (Figure 3). Based on the root branches of the tree and their bootstrap values, 126 of the 140 strains were divided into eight distinct clusters. The remaining 14 strains, except for *B. bifidum* M203F02M632, were grouped into several small clusters containing two to five individual strains. An analysis by geography showed that the strains located in clusters A, C, D, E, and F were all isolated from China. The strains located in clusters A, C, and E were all isolated in this study, indicating that these newly isolated strains have greatly expanded the known genetic diversity of *B. bifidum*. Furthermore, all isolates within cluster G were isolated in Western countries. These findings indicate that the phylogeny of *B. bifidum* may be related to the geographical features of its isolation source and the number of strain-specific genes has a certain relationship with the niche in which they are located.

### 3.4. Identification of CRISPR-Cas Systems and Prophages in B. bifidum

**Figure 4:**
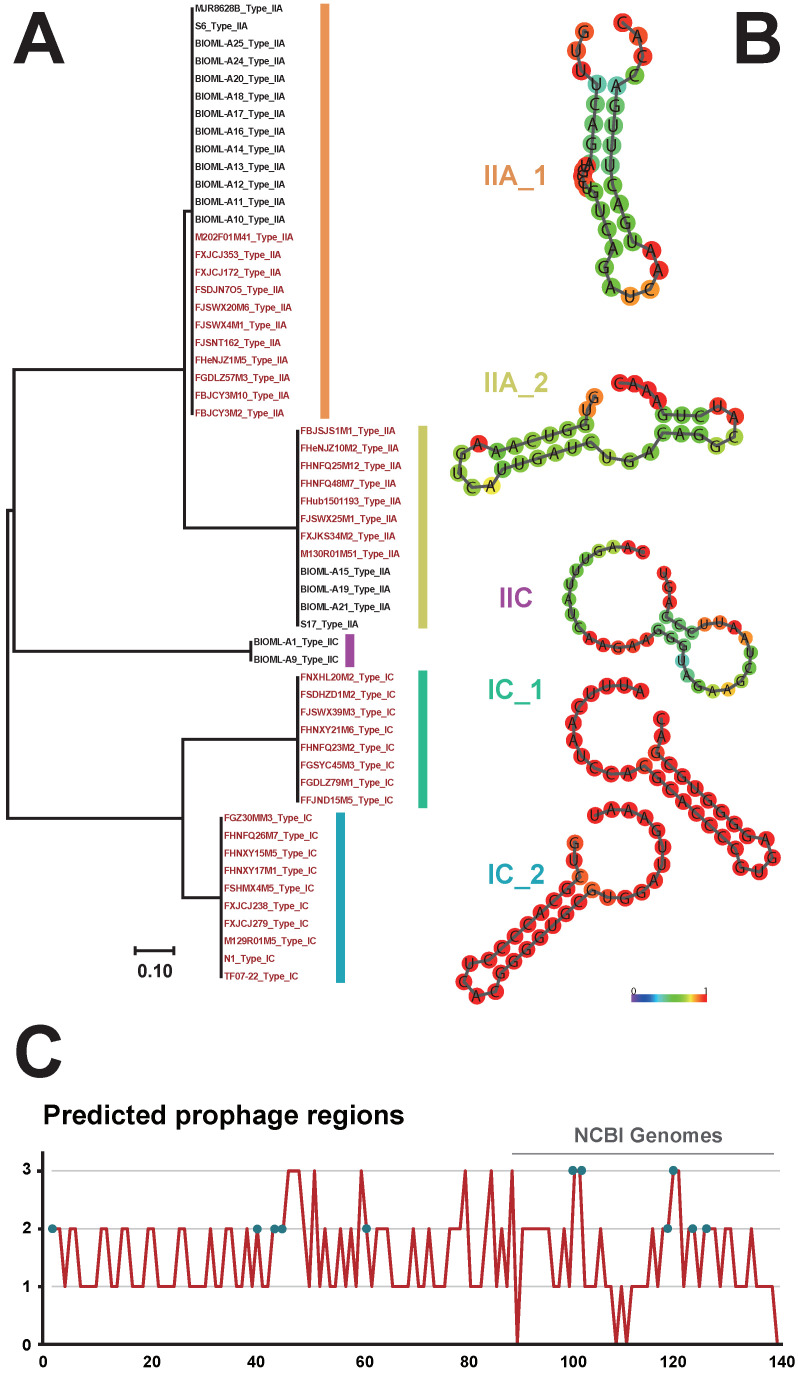
The phylogenetic tree based on direct repeat sequences (**A**), the color bars represent different subtypes of CRISPR-Cas systems in *B. bifidum* genomes. The secondary structures of the repeat sequences in *B. bifidum* strains (**B**), the color in each circle represents the frequency of the amino acid with a gradient from blue (low) to red (high). The prophage regions were identified in *B. bifidum* strains (**C**).

The CRISPR-Cas system confers adaptive immunity to bacteria to resist the insertion of foreign genes, and this system has been gradually implemented for gene editing in bacteria [43]. To provide insights into the diversity of the CRISPR-Cas system in *B. bifidum*, we identified CRISPR-Cas loci and characterized the architecture of each subtype. Overall, 40% (56/140) of the *B. bifidum* genomes encoded CRISPR-Cas systems (Table S2), with type II systems (27%) being more prevalent than type I systems (13%). Based on the presence of signature Cas proteins, we identified 36 strains with a type IIA system, and two strains with a type IIC system. All type I-positive strains had the IC system subtype. Notably, this is the first time that the type I system has been identified in *B. bifidum*. Phylogenetic analysis based on direct repeat sequences was then performed to classify the CRISPR-Cas systems in *B. bifidum* in more detail (Figure 4A). The resulting phylogenetic tree had five major branches. Subtypes IIA and IC were represented by two different branches, whereas subtype IIC had a separate branch, suggesting that the repeat sequences of the type IC and IIA systems are variable. By predicting the secondary structures of the repeat sequences, it was found that both types/subtypes had a typical stable stem-loop structure (Figure 4B). Specifically, for type IIA_1, there were two loops located at the ends of the repeat sequence. In contrast, type IIA_2 contained one additional loop located in the middle of the structure. In addition, the number of spacers in *B. bifidum* was variable, ranging from 38 to 190, thus illustrating that these CRISPR-Cas systems retain immunity memory [43].

As CRISPR-Cas systems may effectively prevent the integration of prophages [44], the 140 *B. bifidum* genomes were scanned for prophage identification. Overall, 215 prophage regions were identified in 136 genomes (Table S3 and Figure 4C); however, none of these were classified as an “intact prophage.” These prophage fragments were incomplete and did not encode functional forms, indicating that functional prophages were virtually absent from *B. bifidum*. Moreover, there was no significant difference in the number of prophage fragments carried by CRISPR-positive and -negative strains (Figure S2). Although the repeat sequence was usually highly conserved throughout the locus, polymorphisms could be observed, notably for the terminal repeat.

### 3.5. Distribution of Bacteriocin Operons in B. bifidum

**Figure 5:**
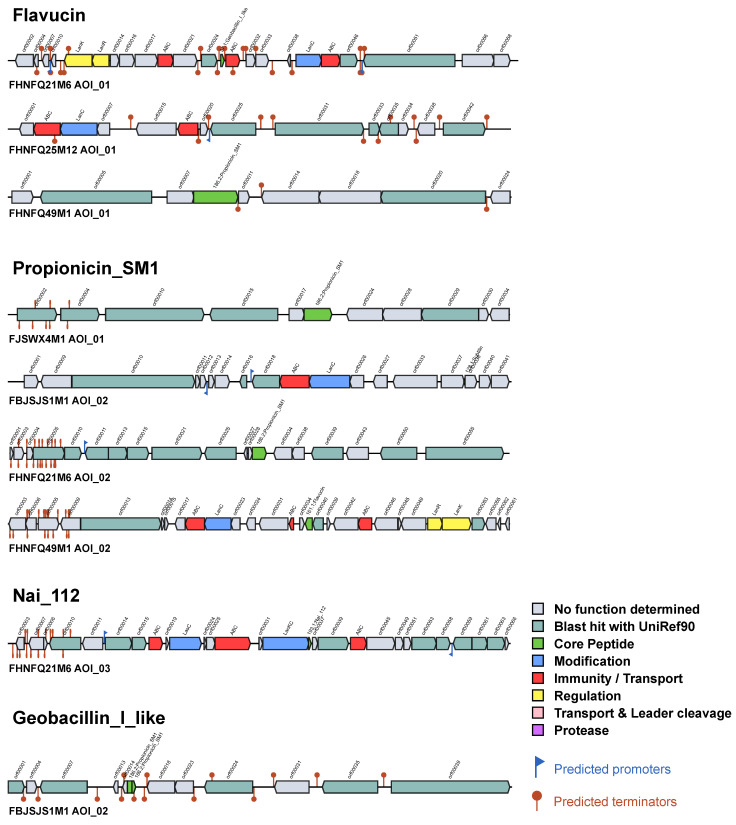
The potential bacteriocin operons identified in five *B. bifidum* genomes.

Bacteriocin is an antimicrobial peptide produced by bacteria. Bacteriocin-producing lactic acid bacteria can effectively inhibit the growth and reproduction of competing bacteria and thus occupy a dominant niche [45]. In this study, only nine potential bacteriocin operons were identified in five genomes (Figure 5). *B. bifidum* synthesizes four bacteriocins, including flavucin, propionicin, N-acetylimidazole, and geobacillin, which belong to class I or II. However, four of the nine operons appeared to be incomplete because they did not contain specific ATP-binding cassette transporters. Additionally, the abundance of *B. bifidum* in the human gut gradually decreases with the age of the host [1], and these five bacteriocin-positive *B. bifidum* strains were isolated from adult feces. Therefore, we speculated that *B. bifidum* may enhance its ecological adaptability by producing bacteriocins.

### 3.6. Carbohydrate Utilization Capacity and Genotype Binding Analysis

As previously mentioned, several *B. bifidum* strains have been reported to utilize host-derived glycans. To determine whether this special carbohydrate utilization capacity is ubiquitous across the species, a local BLAST alignment, with *B. bifidum* PRL2010 as the reference, was performed to identify homologous genes associated with mucin utilization in the 85 isolated strains. As shown in Figure S3, all genes related to mucin metabolism, including those encoding β-N-acetylhexosaminidase, 1,2-A-L-fucosidase, α-1,3/4-fucosidase, 1,3-β-galactosyl-N-acetylhexosamine phosphorylase, endo-α-N-acetylgalactosaminidase, β-galactosidase, lacto-N-biosidase, and exo-α-sialidase, were present in all 85 *B. bifidum* genomes.

**Figure 6:**
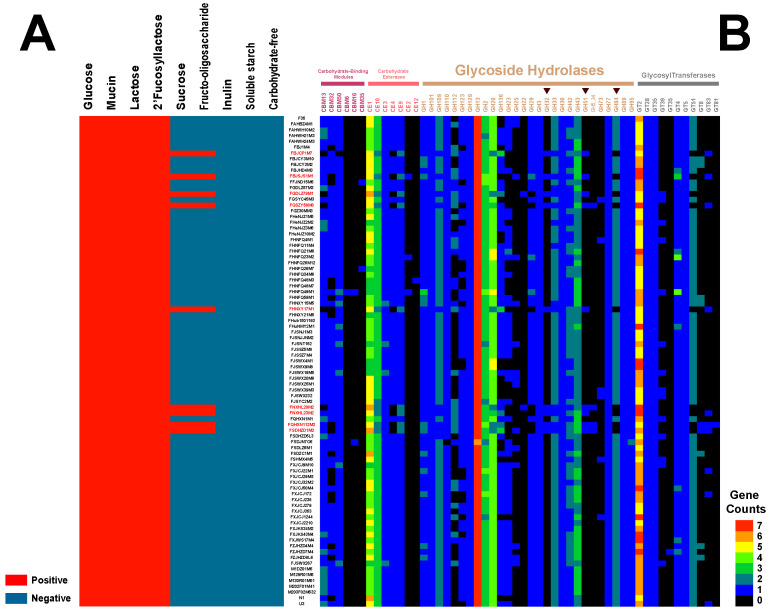
The phenotypes of *B. bifidum* strains that utilized host-derived and plant-derived glycans (**A**). The distribution and number of glycoside hydrolase (GH), carbohydrate esterase (CE), and glycosyltransferase (GT) family genes. Gene copy number was indicated by color ranging from dark (absent) to red (**B**).

To verify the genotypes and phenotypes of *B. bifidum* strains that utilized host-derived and plant-derived glycans, different carbon sources were tested individually. As shown in Figure 6A, all tested strains utilized three host-derived glycogens (lactose, 2′-fucosyllactose, and mucin). In contrast, only a few strains utilized fructo-oligosaccharide and sucrose, and none of the tested strains utilized inulin or soluble starch. These results indicated that *B. bifidum* has the universal capacity to utilize host-derived glycogens, but it does not utilize plant-derived glycogens.

In silico predictions of CAZys were performed for the 85 *B. bifidum* genomes to explain the difference in glycogen utilization by *B. bifidum* strains from different sources. Fifty-two types of CAZys were identified, including 28 GHs, 11 GTs, 7 CEs, and 6 CBMs, and 29 types of CAZys were present in more than 90% of the strains tested (Figure 6B). The ubiquitous GH95 family of genes (α-1,2-L-fucosidase [EC 3.2.1.63] and α-L-galactosidase [EC 3.2.1.22]) in *B. bifidum* explained its phenotype of lactose/2′-fucosyllactose utilization. Notably, we identified GH32 family genes (fructosyltransferases related to fructan and sucrose) in the nine strains that utilized sucrose and fructo-oligosaccharide, while this family of genes was not present in other *B. bifidum* strains. Additionally, these nine strains carried GH51 (endo-β-1,4-xylanase [EC 3.2.1.8], β-xylosidase [EC 3.2.1.37], α-L-arabinofuranosidase [EC 3.2.1.55]) but lacked GH84 (N-acetyl β-glucosaminidase [EC 3.2.1.52]), whereas the other 76 *B. bifidum* strains carried GH84, but lacked GH51. Therefore, these strains have the potential to utilize other plant-derived glycans. Upon tracing the isolation sources of these nine strains, it was found that they were all isolated from adult feces. Therefore, we propose that the ability of *B. bifidum* to use carbohydrates was strain-specific. Some *B. bifidum* strains have developed the capacity to metabolize plant-derived glycogens to adapt to the adult intestinal environment.

### 3.7. Antibiotic Resistance Genotype and Phenotype Analyses of B. bifidum

**Figure 7:**
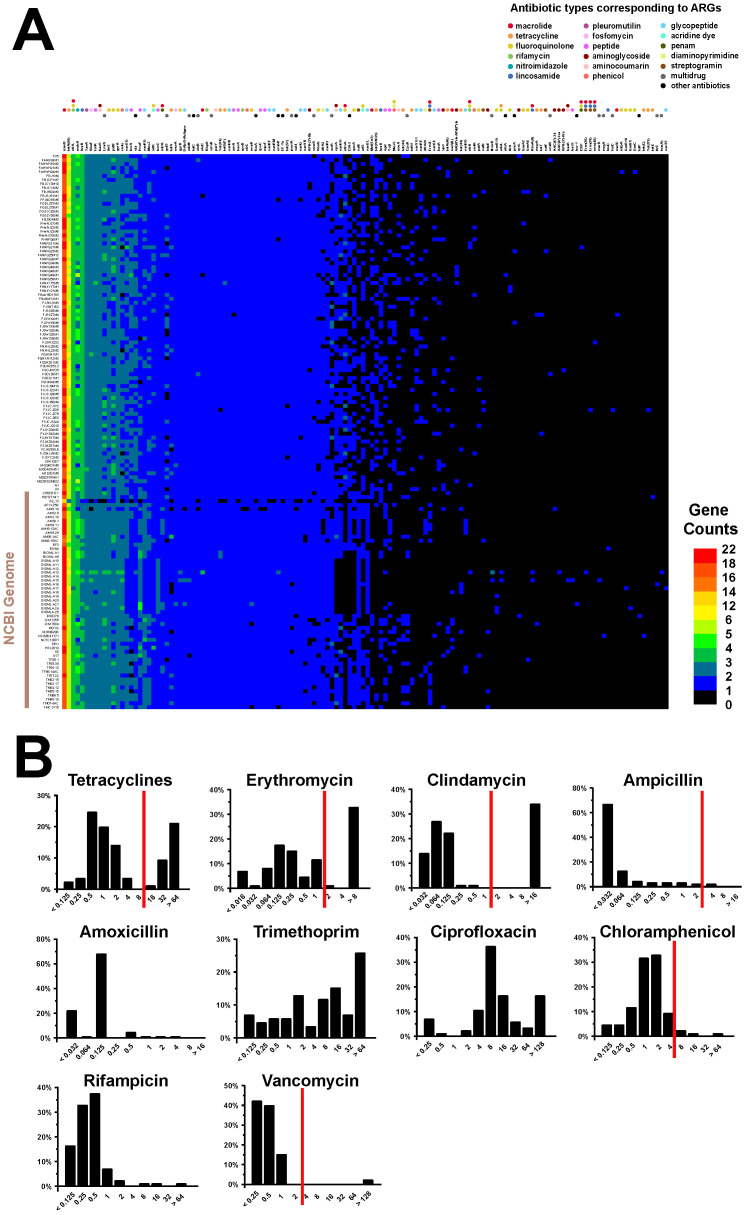
The distribution and the number of potential ARGs in 140 *B. bifidum* genomes. Gene copy number was indicated by color ranging from dark (absent) to red (**A**). The proportions of strains with different MIC values (**B**). The red lines represent microbiological breakpoints recommended by the EFSA.

To evaluate the safety of *B. bifidum*, based on antibiotic resistance, and to provide a reference for future applications in the probiotics industry, 140 *B. bifidum* genomes were scanned to determine whether they harbored potential ARGs. As shown in Figure 7A, each strain carried an average of 106 ARGs, belonging to 129 different types. Fifty-eight of these ARG types, covering a range of common clinically used antibiotics, such as macrolide, tetracycline, fluoroquinolone, rifamycin, nitroimidazole, lincosamide, fosfomycin, and (glyco) peptide, were carried by more than 90% of the genomes tested. Of the remaining 81 types of ARGs, 57 were present in less than 10% of the strains tested, whereas the other 24 types were unevenly distributed in *B. bifidum* strains.

We then measured the minimum inhibitory concentration (MIC) values of 10 antibiotics for the 85 isolated *B. bifidum* strains (Table S4). The proportions of strains with different MIC values are shown in Figure 7B. Based on the breakpoint values of six antibiotics for the genus *Bifidobacterium,* as recommended by the European Food Safety Authority, most of the *B. bifidum* strains were found to be sensitive to ampicillin, chloramphenicol, and vancomycin. Twenty-nine strains were resistant to either erythromycin or clindamycin, and 27 of these strains were resistant to both antibiotics. This indicated that a substantial proportion of *B. bifidum* strains have developed co-resistance to erythromycin and clindamycin. Similarly, approximately 30% of the strains showed tetracycline resistance. For the other four antibiotics without any reference values, analyses of the distribution ranges of MIC values indicated that 95% of the strains (82/85) were sensitive to amoxicillin and rifampicin. Trimethoprim and ciprofloxacin were the two antibiotics with the most variability, with different strains showing different sensitivities to these antibiotics. The MIC values of these antibiotics varied from the lowest concentration to the highest.

## 4. Discussion

Among members of the genus *Bifidobacterium*, *B. adolescentis*, *B. animals*, *B. dentium*, and *B. pseudolongum* are considered to be cosmopolitan species, whereas *B. bifidum* displays a more singular lifestyle and has been specifically identified in the gut of breast-fed infants. In this context, previous comparative genomics studies have concluded that *B. bifidum* is a relatively conservative species, with limited niche adaptability, low genetic diversity, and an open pan-genome. However, the development of genomic tools and the increased number of available sequences have facilitated further analyses of the genomic diversity and function of *B. bifidum*. In this study, genomes of 85 strains isolated from the feces of Chinese infants and adults, combined with 55 publicly available genomes, were used to perform a more comprehensive comparative genomic analysis. We found that *B. bifidum* is not conservative, as previously reported, and a potential mechanism for its adaptation to the adult intestinal environment may exist in some strains. Moreover, new insights into phylogenetic correlations, mobile genetic elements, and antibiotic resistance of *B. bifidum* from this study expand our knowledge of this species.

The average genome size of the 140 *B. bifidum* strains was 2.17 Mb, which is consistent with previous reports [13]. Our genome-wide study of *B. bifidum* showed a trend of the gradual opening up of the genome, suggesting that *B. bifidum* has an open pan-genome and that its pan-genome will increase if more genomes are analyzed and more novel gene families are identified. Moreover, a more open pan-genome implies that gene exchange is higher within the species. These data support the hypothesis that the relative size and content of the pan-genome are potential indicators of the genetic plasticity and environmental adaptation potential of the species. In addition, by annotating the core genes, the defense mechanisms and general functions of the species were predicted.

CRISPR loci, which are present in the genomes of a large number of lactic acid bacteria, provide acquired immunity against foreign genetic elements [44]. However, few research studies have focused on CRISPR in *B. bifidum*. In this study, we specifically analyzed the CRISPR-Cas system in all 140 strains of *B. bifidum* and found that 40% of the strains contained a complete system. This implied that *B. bifidum* is a good candidate for gene editing and the cleavage of lytic bacteriophages in the food industry. The incomplete CRISPR-Cas loci in the remaining strains may be due to genetic recombination, loss of the ability to acquire other CRISPR loci, or the incomplete assembly of the genomes of these strains. The diversity of the Cas protein was significant, but the CRISPR-Cas system could be easily classified (type I–III) by identifying the characteristic proteins (Cas3, Cas9, and Cas10) encoded in the genomes. We found that the type IC, type IIA, and type IIC systems were widespread across *B. bifidum* strains. It is worth noting that the type I system was detected for the first time in *B. bifidum* in this study and was present in 13% of the strains tested. These data provide a new perspective for future investigations of genome diversity and the CRISPR-Cas system in *B. bifidum*.

Screening for bacteriocin in vitro is complex and difficult to achieve, whereas in silico analysis using BAGEL to identify potential bacteriocin operons is generally a more feasible technique. In this study, only nine potential bacteriocin operons, including flavucin, propionicin SM1, Nai_112, and geobacillin_I, were identified in five *B. bifidum* genomes. There has been little research on bacteriocins produced by *B. bifidum*. *B. bifidum* NCFB 1454 has been reported to have the ability to produce bifidocin B [46]. Flavucin is produced by *Corynebacterium lipophiloflavum* DSM 44,291 and displays high antimicrobial activity [47]. Propionicin SM1 is a bacteriocin isolated from *Propionibacterium jensenii* DF1 [48]. Nai_112 is a glycosylated class III lanthipeptide produced by an *Actinoplanes* sp. strain with potent bioactivity against nociceptive pain [49]. Geobacillin I is isolated from the thermophilic bacterium *Geobacillus thermodenitrificans* NG80-2 [50]. The presence of bacteriocin facilitates survival in complex environments and provides bacteria with a competitive advantage [51]. Bacteriocin production may confer *B. bifidum* with a competitive advantage in the intestine and increase its potential for use as a probiotic.

Through direct regulation or indirect modulation via the host microbiota, *B. bifidum* plays an impressive role in attenuating both GI diseases and diseases in remote tissues [12]. There are multiple *B. bifidum* strains with different host origins, and many of the probiotic functions of *B. bifidum* are strain-dependent. Therefore, it may be advantageous to combine different strains of *B. bifidum* to maximize their beneficial effects. However, caution should be taken when drawing inferences based on in silico predictions. Thus, future in vivo analyses performed in murine models or clinical trials will be needed to validate the results described in this study.

## 5. Conclusions

A comparative genomic analysis of 140 strains of *B. bifidum* isolated from different niches was performed in this study. The comparative genomic results show that the number of strain-specific genes has a certain relationship with the niche in which they are located. The core functions of *B. bifidum* were concentrated on the utilization of host-derived glyans, which provide molecular support for strains to metabolize multiple sugars. Further literature mining of the carbohydrate metabolism-related genes would improve the prediction accuracy of this method. Additionally, the genetic diversity of *B. bifidum* was mainly reflected in the glycoside hydrolase gene family, bacteriocin operons, antibiotic resistance genes, and CRISPR-Cas loci. These results provide new information and a framework for further investigating the evolution.

## Data Availability

This study generated sequencing data for 85 *B. bifidum* isolates, and all sequence data have been deposited in the National Center for Biotechnology Information.

## Supplementary Materials

The following are available online at https://www.mdpi.com/article/10.3390/genes12101504/s1, Figure S1: Pan-genome, core genes and unique genes among 55 *B. bifidum* genomes accessed from NCBI, Figure S2: Comparison of the number of prophage fragments in *B. bifidum* genomes with or without a CRISPR-Cas system, Figure S3: Homologous genes associated with mucin utilization in the 85 *B. bifidum* isolated strains, Table S1: Specific information of 140 *B. bifidum* genomes involved in this study, Table S2: The distribution of CRISPR-Cas systems among 140 *B. bifidum*, Table S3: Prediction of prophages among 140 *B. bifidum* genomes, Table S4: The minimum inhibitory concentration of 85 *B. Bifidum* to ten antibiotics.
